# Examination of promotive and protective effects on early adolescent prosocial behavior through a bioecological lens

**DOI:** 10.3389/fpsyg.2023.1280346

**Published:** 2023-11-17

**Authors:** Elizabeth J. S. Bates, Lauren M. Berny, Jody M. Ganiban, Misaki N. Natsuaki, Jenae M. Neiderhiser, Daniel S. Shaw, Leslie D. Leve

**Affiliations:** ^1^Department of Counseling Psychology and Human Services, University of Oregon, Eugene, OR, United States; ^2^Department of Psychological and Brain Sciences, George Washington University, Washington, DC, United States; ^3^Department of Psychology, University of California, Riverside, Riverside, CA, United States; ^4^Department of Psychology, The Pennsylvania State University, University Park, PA, United States; ^5^Department of Psychology, University of Pittsburgh, Pittsburgh, PA, United States; ^6^Prevention Science Institute, University of Oregon, Eugene, OR, United States

**Keywords:** prosocial behavior, bioecological model, longitudinal, childhood, parental warmth, promotive effects, protective effects

## Abstract

**Introduction:**

Prosocial behavior during childhood has been associated with numerous positive developmental and behavioral outcomes in adolescence and adulthood. Prosocial behavior, which includes cooperation and helping others, develops within a bioecological context. Considering it through such a lens enhances the understanding of the roles of different bioecological factors in its development.

**Methods:**

Using data from a longitudinal study of adopted children and children reared with their biological parents, this paper examined if positive aspects of a child’s bioecological system at age 7 predict prosocial behavior in early adolescence (age 11), and whether these bioecological factors could offset risk due to biological family psychopathology and/or maternal prenatal substance use. The analyses incorporated variables from different levels of Bronfenbrenner’s bioecological model (the individual, microsystem, exosystem, and macrosystem) and examined the promotive, and potentially protective, effect of each contextual factor, while also considering their interplay with biological family psychopathology and prenatal substance use.

**Results:**

Results from linear regression models indicated that the microsystem variable of parental warmth at age 7 had a promotive effect on age 11 prosocial behavior. Further, in addition to its main effect, parental warmth was protective against maternal substance use during pregnancy when children were raised with their biological parent (s). Household type (biological family) and biological family internalizing psychopathology were the only other significant predictors in the model, with each associated with lower prosocial behavior at age 11.

**Discussion:**

Study results extend prior work on the benefits of parental warmth on child outcomes by employing a strength-based, bioecological approach to the development of prosocial behavior during early adolescence and examining “for whom” the effects of parental warmth are most protective.

## Introduction

Prior research has found that prosocial behavior during childhood—which includes cooperation, helping others, generosity, kindness, and accountability (Eisenberg et al., 2006)—is associated with positive relationships with peers (Fowler and Christakis, 2010; Flook et al., 2019), social adjustment in school settings (Layous et al., 2012), reduced risk of developing problem or externalizing behaviors (Wentzel and McNamara, 1999; Flouri and Sarmadi, 2016), better overall mental health (Almedom, 2005), and improved academic performance (Eisenberg et al., 2006; Carlo et al., 2018). Furthermore, prosocial behavior can contribute to children’s well-being by improving their ability to cope in challenging situations and form social connections to facilitate a sense of belonging (Masten, 2021). As such, prosocial behavior is an indicator of and contributor to positive adaptation and resilience, making it important to understand what factors and conditions support its development.

Eisenberg et al. (2006) highlighted the importance of understanding how biological factors and environmental influences throughout childhood jointly shape prosocial behavior development. This perspective has recently been reinforced by the National Scientific Council on the Developing Child (NSCDC, Boyce et al., 2021; Shonkoff et al., 2021), which emphasized considering the impact of children’s biological characteristics on development within the context of structural and social factors. Using Goodman’s (2001) definition of prosocial behavior as being considerate, caring, kind, and helping out and guided by the perspectives of Eisenberg and the NSCDC, the current study takes a strength-based approach to examine the longitudinal associations of positive bioecological influences on the development of behavioral dimensions of prosocial actions from middle childhood to early adolescence. Employing a bioecological lens (Bronfenbrenner, 1974; Bronfenbrenner and Morris, 2007), we also assess whether these precursors are protective, such that in the context of adversity, they prospectively predict child prosocial behavior, offsetting the deleterious effects of exposure to adversity (Masten et al., 2021).

### Bronfenbrenner’s bioecological model and development of prosocial behavior

Within Bronfenbrenner’s bioecological model, influences on a child’s development are conceptualized as multiple interacting levels encircling the child themself at the center. The individual level includes biological characteristics like sex and biological pathophysiology, genetic makeup and predispositions, and child temperament. The individual is then surrounded by three additional levels: (1) the microsystem consisting of their immediate environment of family, peers, and teachers; (2) the exosystem consisting of their extended environments such as neighborhoods and communities; and (3) the macrosystem consisting of broader societal and cultural influences (Bronfenbrenner, 1974; Bronfenbrenner and Morris, 2007). This paper focuses on positive aspects of the child’s bioecological system and their contributions to children’s prosocial behavior, with a focus on specific components in the individual level, microsystem, exosystem, and macrosystem that are theorized to be positively associated with early adolescent prosocial behavior.

#### Individual level: positive affectivity

In context of this study, the individual level of Bronfenbrenner’s model relates to children’s innate traits and characteristics that influence their prosocial development. Positive affectivity is a dimension of temperament that is known to be heritable (Rothbart et al., 2000) and can be considered to fall under Bronfenbrenner’s individual level of the bioecological model. Children with greater positive affect may elicit more positive responses from peers and adults, thus providing them with opportunities for socialization and modeling to develop prosocial behavior. For example, in a review of literature on positive affectivity, Davis and Suveg (2014) conclude that older children and adolescents with positive affectivity have improved social functioning and more social relationships. Additionally, the resulting positive peer interactions and adult engagement can reinforce the child’s prosocial behavior (reflective of a positive feedback loop) and encourage further development (Eisenberg et al., 2006, 2009; Borowski et al., 2021). The current study builds on this prior research to examine middle-childhood positive affectivity (age 7) as a prospective predictor of prosocial behavior in early adolescence (age 11).

#### Microsystem: parental warmth

A child’s microsystem, which encompasses their immediate environment of family, peers, and teachers, shapes prosocial behavior through unique contributions from each person of influence and their relationship with the individual child. In many cultures, positive family influences during childhood are associated with adolescent prosocial behavior (Malonda et al., 2019; Pastorelli et al., 2021). Parental warmth, which encompasses emotional nurturance and sensitive and responsive parenting, is one such family influence identified by prior research as being predictive of prosocial tendencies in children (Eisenberg et al., 2006; Padilla-Walker et al., 2018; Spinrad et al., 2019). Several cross-sectional studies have shown the positive associations between parental warmth and youth prosocial behavior, measured in ages 9–14 years old (Zhou et al., 2002; Carlo et al., 2018). In a cross-lagged model with longitudinal data from mother/child dyads, Luengo Kanacri et al. (2021) found that maternal warmth experienced at ages 9–13 predicted prosocial behavior at ages 12–15. Another study found that, even when controlling for potential genetic influences on parenting behavior, parental warmth and the related family context were positively associated with prosocial behavior in children at ages 3, 4, and 7 (Knafo and Plomin, 2006).

Pastorelli et al. (2021) proposed three theoretical explanations for the association between parental warmth and adolescent prosocial behavior. First, applying Social Learning Theory (Bandura, 1977), a child may model a parent’s care and helping of other by emulating that behavior (Eisenberg et al., 2016). Second, parental warmth can create a positive rearing environment for children, in which children feel more deeply connected to individuals and develop care and concern for others as early as infancy (Eisenberg et al., 2009). Third, parental warmth can build a positive parent–child relationship that promotes joint emotional self-regulation, perspective-taking, and empathy, which results in a reciprocal show of kindness and caring beginning in preschool and extending through adolescence (Eisenberg et al., 2006; Pastorelli et al., 2021). Thus, we anticipated that when examined prospectively and in the context of multiple other bioecological factors, parental warmth would emerge as a salient predictor of prosocial behavior in early adolescence.

#### Exosystem

A child’s exosystem includes social structures and contexts that can directly influence a child’s prosocial behavior and indirectly shape their prosocial development by influencing factors in the child’s microsystem. A large body of work has examined neighborhood characteristics, specifically the role of neighborhood structure and social communities, in influencing youth behavior (see review by van Ham and Manley, 2012; Chetty and Hendren, 2018; Chyn and Katz, 2021). Positive aspects of the exosystem, such as neighborhood cohesion, efficacy, and safety, are theoretically associated with prosocial behavior (Lenzi et al., 2012; O’Brien and Kauffman, 2013). The current study focused on two specific positive elements of neighborhoods to examine potential promotive and protective effects through social-interactive and environmental mechanisms (van Ham and Manley, 2012): neighborhood involvement and neighborhood safety.

##### Neighborhood involvement

Neighborhood involvement is considered a key component of social cohesion and is the foundation of neighborhood collective efficacy (Gerell, 2015). Neighborhood collective efficacy, which has been defined as neighborhood residents’ ability and willingness to improve their neighborhoods, has been shown to influence social patterns within a community. This dimension of neighborhood collective efficacy, which includes building relationships and neighborhood engagement, has also been shown to support prosocial behavior for 10–18 year olds (Lenzi et al., 2012; O’Brien and Kauffman, 2013; Gerell, 2015). Lenzi et al. (2012) highlighted the benefits of perceived social cohesion in promoting adolescent prosocial behavior in a study of adolescents ages 11–15. It is possible that neighborhood and related “nurturing environments,” as referred to in other studies (Biglan and Hinds, 2009), benefit youth through socialization and creating a culture of kindness and support (Lenzi et al., 2012). When youth experience greater social support, it increases their tendency to develop the social norms of their community and act with collective efficacy. Therefore, a neighborhood with more group involvement, group support, and social cohesion can beget greater prosocial tendency among adolescents living within it (Lenzi et al., 2012).

##### Neighborhood safety

A majority of research on the association between neighborhood safety and child behavior focuses on lack of safety and risky environments given their association with antisocial and delinquent behavior, with recent work highlighting prosocial development in risky contexts (see Taylor and Carlo, 2021). Fewer studies have examined the association between positive neighborhood attributes and prosocial behavior, yet some recent research has shown that safer environments have been associated with greater prosocial behaviors in youth. Specifically, in a cross-sectional study with a diverse, nationally representative U.S. sample of youth ages 9–10 and their caregivers, Memmott-Elison et al. (2021) found an indirect link between parent-perceived neighborhood safety and youth prosocial behavior via parental mental health and family conflict. This indirect association between neighborhood safety and prosocial behavior was similar across ethnic and racial groups and may reflect a degree of interplay between exosystem (i.e., neighborhood) and microsystem (i.e., household) factors. The current study adds to literature in this area by exploring the longitudinal, direct association between neighborhood safety and early adolescent prosocial behavior.

#### Macrosystem

Macrosystem-level factors include societal or cultural influences on a child’s prosocial behavior, such as religious affiliations and financial security, as they dictate norms, values, ideas, and ways of interacting with the world.

##### Religious affiliations

Religion is considered a cultural influence as it dictates norms, values, and ideas that influence the way one interacts with the world, making it a core element of Bronfenbrenner’s macrosystem level influences on child development. The importance of religious or spiritual beliefs can guide certain actions and interactions with others (Malhotra, 2010). Prosocial behavior appears throughout religious doctrine, including as a foundational tenent in Judaism, Christianity, and Buddhism (Eisenberg et al., 2006). The saliency of an individual’s religious affiliation and the associated moral beliefs have been connected with generosity, cooperation, and other attributes of prosocial personalities in children and adults in cross-sectional studies (Furrow et al., 2004; Saroglou et al., 2005; Malhotra, 2010), yet there is a dearth of longitudinal research exploring associations with prosocial behavior outcomes in youth.

##### Financial security

Financial security relates to a household’s financial standing in relation to societal level factors such as costs of basic needs (e.g., food, housing) and household income. The association of financial security with a child’s prosocial behavior is a result of the interaction of broader economic, political, and societal structures (e.g., inflation, wages, work stress, and employment opportunities) with the more proximal neighborhood-level (exosystem) and family-level factors (microsystem) as well as the individual child themselves (Li et al., 2008). Few studies have examined the prospective relationship between financial security and prosocial behavior; rather, existing literature has primarily focused on the longitudinal effects of financial insecurity/strain and material hardship on increasing risk of problem behavior or decreasing levels of prosocial behavior (e.g., Mazza et al., 2017). One suggested mechanism is that economic strain decreases capacity for empathy as an adaptive response to scarcity, which leads to decreased prosocial behavior (Jiang et al., 2020). Another study, grounded in the Family Stress Model, illustrates how family economic stress can negatively impact youth prosocial behavior (Xiao et al., 2023). Specifically, in a cross-sectional study with youth ages 11–14, Xiao et al. (2023) found that economic pressure, also referred to as financial stress or strain, was negatively associated with two types of prosocial behavior (dire and emotional types). We apply this logic to examine financial security by hypothesizing that individuals in families with greater financial security are better positioned to give more social and emotional support to those around them given their lower strain and competition for resources.

### The potential for protective effects

The sections above described the potential promotive role of a range of bioecological factors in predicting adolescent prosocial behavior. In addition, these same bioecological factors could have protective effects depending on the environment of the child (e.g., amount of adversity in the environment; Masten et al., 2021). Using the resilience model outlined by Masten et al. (2021), promotive effects lead to improved outcomes regardless of the environment or any adversity present. In comparison, protective effects lead to improved outcomes in an adverse or risky context, thereby enabling adaptation. In this sense, protective effects can buffer the deleterious effects of risk factors or adversity (Masten et al., 2021). The strength of the protective effect can vary depending on how much adversity or risk exists in the environment. For example, in a longitudinal parent-offspring study of children 18 months to 8 years of age, Leve et al. (2022) found that structured parenting was protective and buffered risk associated with biological family psychopathology on child behavior problems for children with high biological family psychopathology risk. However, for children with low biological family psychopathology, structured parenting was not protective but rather associated with more child behavior problems. In a study focused on protective effects in the context of prenatal substance use, Bada et al. (2012) examined the roles of a variety of protective factors that can mitigate behavioral outcomes associated with maternal cocaine use during pregnancy. In a longitudinal cohort study of children born with prenatal cocaine or opioid exposure, the presence of protective factors throughout childhood improved internalizing and externalizing behavior problems measured from ages 5–15 years old. The protective factors included self-reported resilience and caretaker involvement measured at ages 11–15 years and family resources measured at age 5.5 years and 9 years. When in a persistently high-risk environment, including child, family and community risk factors, a high protective index (i.e., greater exposure to numerous protective factors) continued to have a protective effect and offset the predicted association between prenatal drug exposure and increased problem behavior. The current study sought to examine the potential protective effects of the aforementioned bioecological factors on prosocial behavior against risk conferred by two factors: biological family psychopathology and prenatal substance use.

### Risk factors: biological family psychopathology

Biological family psychopathology, including internalizing and externalizing symptoms, are associated with decreased prosocial behavior and increased antisocial behaviors in children (Kim et al., 2009). Biological parents’ internalizing and externalizing behaviors have been linked with similar internalizing and externalizing behavior symptoms in their children, even when the child is reared in an adoptive home with non-genetically related rearing parents (Kerr et al., 2013; Marceau et al., 2019). This is indicative of the heritable role of biological family psychopathology, in addition to the environmental transmission of psychopathology across generations. Maternal stress and anxiety, for example, can be inherited as a behavioral predisposition (i.e., intergenerational genetic transmission), and can also be biologically embedded through stress-elicited inflammatory responses that change multiple systems, potentially occurring prenatally (Coussons-Read, 2013; Dunn et al., 2019). Similarly, inhibitory control deficits, which are seen as deterrents for prosocial peer interactions (Eisenberg et al., 2006), have been shown to be heritable (Polderman et al., 2009), and their effect on social behaviors can be exacerbated by environment factors, such as parental hostility or lack of inhibition (Leve et al., 2019). In families where children are raised by their biological parent (s), the underlying mechanism of behavior transmission could be environmental or genetic in nature, and it is likely both processes are operating. However, in adoptive households when children are not reared with their biological parents, the mechanism linking biological family psychopathology and child behavior can only be the result of genetic or prenatal influences as the biological family is not providing the rearing context. In the current study, the sample includes both children reared in adoptive homes and children reared in biological homes, allowing for an additional examination of whether the associations between biological family psychopathology (and prenatal substance used, described next) and child prosocial behavior may differ across household types.

### Risk factors: prenatal substance use

Prenatal substance use is the second factor that is related to the behaviors of the biological family (the pregnant mother in this instance). Prenatal substance use could lead to reduced child prosocial behavior directly via impacts on fetal development or indirectly via effects on the postnatal rearing environment. Specifically, the well-founded evidence on the harmful effects of prenatal substance use on later child developmental outcomes in emotional regulation and processing (Pechtel and Pizzagalli, 2011; Neiderhiser et al., 2016) may be a result of the prenatal environment from prenatal substance use exposure as well as the postnatal environment related to prenatal substance use exposure. As described above, when children are reared by their biological parents, who provide the rearing environment and the prenatal environment, the underlying mechanism explaining the influence of prenatal substance use on child behavior cannot be distinguished between prenatal or postnatal environmental. However, in adoptive households when children are not reared with their biological parents, links between prenatal substance use and child prosocial behavior are not confounded by the postnatal rearing environment. This current study includes both household types, allowing for further elucidation of the underlying processes of the influence of prenatal substance use.

A majority of research on the association between prenatal substance use, particularly alcohol and opioid use, and child developmental outcomes has focused on the negative social and emotional outcomes (e.g., Kully-Martens et al., 2012). Although less is known about the effects of prenatal substance use on a child’s prosocial behavior, it has been theorized that disrupted development of emotion regulatory processes can also impair regulation of social behavior, thus decreasing prosocial tendencies (Eisenberg et al., 2006). de Water et al. (2021) examined the theorized association between prenatal substance use and decreased prosocial tendencies and found that children and adolescents ages 8–16 years old with prenatal alcohol exposure exhibited less parent-reported prosocial behavior and more parent-reported antisocial behavior than their same age peers who were not exposed to alcohol *in utero*. Given the potential for adverse associations between prenatal substance use and children’s prosocial behavior, we also sought to examine whether there are positive elements of the bioecological model that can offset its role. For example, as referenced above when discussing protective factors, Bada et al. (2012) found that protective factors within children’s individual, family, and community levels mitigated behavioral problems associated with maternal substance use during pregnancy. The current study builds off the findings of the protective effects in adverse conditions found by Bada et al. (2012) and examines the role of promotive and protective effects on prosocial behavior in early adolescence.

### The current study

**Figure 1:**
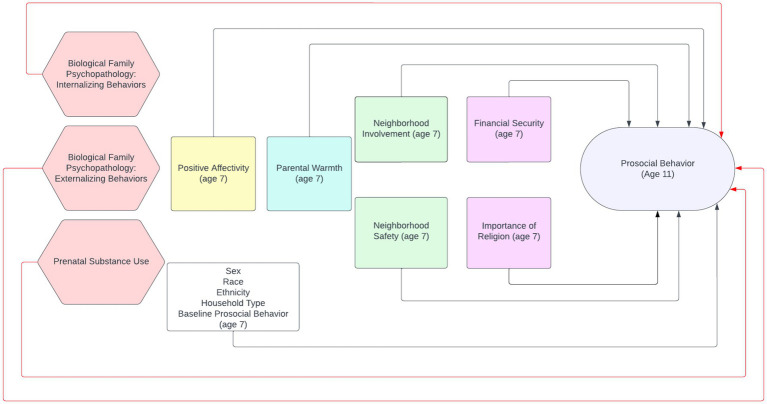
Diagram illustrating the conceptual model that incorporates variables across the child’s bioecological context and risk factors. The diagram is color-coded according to risk factors (red: biological family psychopathology and prenatal substance use) and each level of Bronfenbrenner’s bioecological model: individual (yellow); microsystem (teal); exosystem (green); macrosystem (purple). Covariates are included in the white box.

The current study extends the understanding of bioecological influences on child development by focusing on longitudinal associations between positive bioecological factors assessed in middle childhood and prosocial behavior in early adolescence. We take a strengths-based approach by examining promotive and protective factors in the context of biological family psychopathology and prenatal substance use risk factors. Using data from a longitudinal study of adopted children and their biological siblings raised by the birth parent (s), this paper tests whether positive elements of the bioecological model measured at child age 7 promote prosocial behavior measured at age 11, and if these factors have a protective effect on prosocial behavior and can offset risk from biological family psychopathology and/or prenatal substance use. We further examine whether these associations vary by home type (adoptive or biological home) given that in the latter, genetic, prenatal, and environmental contextual influences emanate from the same parent (s), whereas in adoptive homes, genetic and prenatal influences originate from the biological family but the environmental context the child is raised in is guided by the adoptive family. The analyses incorporate variables from all levels of Bronfenbrenner’s bioecological model (the individual, microsystem, exosystem, and macrosystem), allowing for a comprehensive examination of promotive and protective effects across the child’s bioecological context and a consideration and a consideration of their interplay of their interplay with biological family psychopathology and prenatal substance use (Figure 1).

The primary study aim was to examine the main effects of positive factors on children’s subsequent prosocial behavior and assess their promotive role. A secondary aim was to examine the interactive effects between the significant promotive factors in the context of exposure to biological family psychopathology or prenatal substance use. In other words, to examine protective effects of the identified promotive factors. A third aim was to use the study’s adoption and biological family design to test whether associations in the first two aims differed by household types, given that children raised in biological homes can be influenced by a confluence of prenatal, genetic, and post-natal factors from their biological parents, whereas the influences from those factors are more delineated in adoptive homes. For adoptees, the data on prenatal and biological influences originate from their birth parents, who are not rearing the adoptee, whereas data on family and contextual influences are reported by the adoptee and/or their genetically unrelated, rearing parents. For the biological siblings, all data originate from the child and their biological parents who share genetic make-up. In addition, the ecological contexts were different across households, with the adoptive households, on average, having greater socioeconomic advantage (i.e., income and educational attainment; Natsuaki et al., 2019). As a result, this design creates opportunities to better understand the nature of the complex pathways that influence children’s prosocial behavior, and in particular, to examine if any promotive or protective effects vary by household type.

We asked four research questions, utilizing data on promotive factors at age 7 and prosocial behavior at age 11: (1) Do positive factors across multiple levels of the bioecological model at age 7 have *promotive effects* on prosocial behavior at age 11? We hypothesize that all bioecological factors at age 7 would have a significant association with prosocial behavior at age 11; (2) Do these positive bioecological factors have a *protective effect* on the relationship between biological family psychopathology and/or prenatal substance use and early adolescent prosocial behavior? We hypothesized that identified promotive factor (s) would be protective in the context of both biological family psychopathology and prenatal substance use based on existing work on buffering effects of intergenerational transmission of behavior (Bada et al., 2012; Neiderhiser et al., 2016; Leve et al., 2022); (3) Do the potentially protective effects tested in Hypothesis 2 vary across household type (biological household or adoptive household)? For example, does the association between biological family psychopathology or prenatal substance use and the identified promotive factors on prosocial behavior differ between the adoptive sample vs. children reared with their biological parent (s)? We hypothesized that the protective effects examined in Hypothesis 2 would be more pronounced in biological households than in adoptive households; and (4) Do the bioecological factors and the two risk factors measured in Hypotheses 1–3 significantly differ between biological households and adoptive households? This is an exploratory research question with the goal of better understanding and contextualizing the results from Hypotheses 1–3. However, we anticipated there will be significant differences between environmental factors present in biological households compared to adoptive households, particularly in ecosystem and macrosystem variables based on previously identified socioeconomic differences between the two groups (Natsuaki et al., 2019).

## Methods

### Participants

This study analyzed secondary data collected from participants in the Early Growth and Development Study (subsequently referenced as EGDS; Leve et al., 2019). The EGDS study includes 561 adoptive families, recruited from 45 adoption agencies in 15 U.S. states. A complete description of the EGDS methodology is outlined in Leve et al. (2019). A summary of the sampling methodology is as follows. Participants were identified by agency liaisons who screened families for the study’s eligibility criteria: (a) the adoption placement was domestic, (b) placement occurred within 3 months postpartum, (c) the infant was placed with an adoptive family that was not biologically related to the child, (d) there were no known major medical conditions such as extreme prematurity or extensive medical surgeries, and (e) the birth and adoptive parents were able to understand English at an 8th grade level. All types of adoptive families were eligible for study enrollment (e.g., same-sex parents, single parents, and hearing-impaired parents). At a later stage, 217 biological siblings of the adoptees, who were raised by their biological parent (s), were recruited into the study. The eligibility criteria included: (a) birth mother enrolled in EGDS between 2003 and 2009 following the birth of the EGDS adoptee, (b) birth mother was parenting a biological sibling of an EGDS adoptee, and (c) this biological sibling was born between 2005 and 2012. These participants were recruited at age 7 from an eligible pool of *n* = 287 children. To assess sampling bias in the recruitment of the original sample of birth and adoptive families, demographic information was compared between triads who participated in the EGDS (*N* = 561 triads) and the eligible nonparticipants (*N* = 2,391 triads available for analysis) using data available from the participating agencies. Minimal systematic sampling biases were found, suggesting that the EGDS sample is generally representative of the population from which it was drawn (see Leve et al., 2013 for details). The analytic sample for the current study included 466 children who completed a questionnaire measuring prosocial behavior at age 11. Eighty-three percent of the analytic sample (*n* = 386) lived with non-genetically related adoptive parents and 17% of the sample (*n* = 80) lived with at least one biological parent. Demographic characteristics of the analytical sample are displayed in Table 1. Four hundred and six participants were from unique families; 60 participants (12.7% of the full analytic sample) shared a biological parent with at least one other participant. Approximately 55% of the children were male. When initially sampled, parents were asked to report on their child’s ethnicity (Hispanic or non-Hispanic) and race (Asian, American Indian/Alaskan Native, Black, White, or Multiple Races). Most of the children were White Non-Hispanic (56.7%), followed by 23.0% who identified as multiple races, 13.1% as Black, and 7.3% as some other race or ethnicity which included individuals who identified as American Indian/Alaskan Native, Asian, or White Hispanic. This latter group (other race or ethnicity) was aggregated for the purpose of model convergence given the small sample sizes for those racial and ethnic groups. Most adoptive mothers and adoptive fathers were White Non-Hispanic (91.3 and 90.6%, respectively); others were Black (2.9 and 0.9%, respectively), multiple races (2.2 and 0.9%, respectively), and some other race or other ethnicity (3.5 and 6.9%, respectively). The majority of birth mothers and birth fathers were White Non-Hispanic (64.2% and 58.1.5%, respectively); others were Black (20.9 and 25.7%, respectively), multiple races (3 and 10.8%, respectively), and some other race or other ethnicity (11.9 and 5.5%, respectively). Some characteristics differed across household types. Whereas the median total annual household income for adoptive families was between $100,001–$125,000, and the median educational attainment for adoptive parents was at least a 4-year college degree, the median total annual household income for biological families was $25,001–$40,000, and their median educational attainment was a high school diploma. All research activities were approved by the Institutional Review Boards at University of Oregon and The Pennsylvania State University. Participating parents provided consent, and all participating children provided assent for the research activities.

**Table 1:** Descriptive statistics of analytic sample (*n* = 466 children).

Variables	*M* or %	*SD*
Child Sex		
Male	55.15	%
Female	44.85	%
Child Race & Ethnicity		
White non-Hispanic	56.65	%
Multiple races	22.96	%
Black	13.09	%
Other race or ethnicity	7.30	%
Household		
Biological parent	82.83	%
Adoptive parent	17.16	%
Prenatal substance use	37.73	%
BF externalizing behaviors	0.09	1.80
BF internalizing behaviors	0.02	1.58
Prosocial age 7	49.06	9.48
Prosocial age 11	8.15	1.71
Positive affectivity	5.85	0.57
Parental warmth	38.01	3.31
Neighborhood involvement	1.86	0.53
Importance of religion	2.03	0.96
Financial security	6.03	1.53
Neighborhood safety	4.45	0.45

*Notes*. Descriptives pooled across 25 multiply imputed datasets. BF = biological family; *M* = mean; *SD* = standard deviation. Prosocial behavior at age 7 was collected with the Social Skills Rating System (SSRS) and prosocial behavior at age 11 was collected with the Strength and Difficulties Questionnaire (SDQ).

### Procedures

Data were collected through online questionnaires and phone interviews. The dependent variable, prosocial behavior, was self-reported by the child at age 11. All predictor variables were collected at age 7, and, with the exception of neighborhood safety, were reported by the rearing parent (s). Neighborhood safety was collected as an observational report from a trained interviewer. When two parent reports were provided, their responses were aggregated and a mean score used. For predictors with only one parent’s report (i.e., in a single-parent home or only one parent participated), the sole parent’s response was used. All of the measures used in the current study are standardized measures that were validated in prior research.

### Measures

#### Dependent variable: prosocial behavior (age 11)

Prosocial behavior was measured at age 11 using five items in the prosocial scale (α = 0.52) from the Strengths & Difficulties Questionnaire (SDQ; Goodman, 1997, 2001) that assessed caring, sharing, kindness, and helping behaviors. Children participated in a phone interview or web survey and rated their level of agreement with each item on a scale of 0 (“not true”) to 2 (“certainly true”). Items were aggregated together to create a composite score with higher scores indicating more prosocial behavior.

#### Positive predictor variables across ecological levels (age 7)

##### Individual-level predictor

The individual-level ecological factor measured at age 7 in the current study was positive affectivity, which was measured using the “smiling and laughter” subscale of the Children’s Behavior Questionnaire (Rothbart et al., 2001; Putnam and Rothbart, 2006). This scale includes 13 items measured on a 1–7 Likert-type scale ranging from “extremely untrue of your child” to “extremely true of your child.” The α range reflects the range of the two parents’ reports. Item scores were aggregated to form a composite score. Mean score of the two parents’ ratings was used when two parents completed this questionnaire (*r* = 0.247, paired observation *n* = 270). Interitem reliability was acceptable (α = 0.79 to 0.85).

##### Microsystem-level predictor

The microsystem promotive factor measured in the current study was parental warmth. Parental warmth toward the child they were rearing (adopted or biological) was measured by parent self-report on the 6-item warmth scale from the Iowa Family Interaction Rating Scales (Melby and Conger, 2001). Parents rated items on a 7-point Likert scale ranging from “always” to “never” with higher scores indicating higher warmth. Items asked about the parent’s warmth to the child, such as “let him/her know you really care about him/her,” “let him/her know that you appreciate his or her ideas or the things he/she does,” “help him/her do something that was important to him/her,” and “tell him/her you love him/her” (Melby and Conger, 2001). Item scores were aggregated, and a mean score of the two parents’ ratings was used when two parents completed this questionnaire (*r* = 0.383, paired observations *n* = 244). Interitem reliability was acceptable (α = 0.81–0.89).

##### Exosystem-level predictors

Community-level protective factors measured in the current study included neighborhood involvement and neighborhood safety. Neighborhood involvement was indexed by parent report using one item from the Dumisha Social Support Scale, a measure in the General Life Satisfaction Scale, rated on a 3-point Likert scale. The item asked parents how involved they were within their neighborhood or community, ranging from “not at all” to “very involved” (Crnic et al., 1983). A mean score of the two parents’ ratings was used when two parents completed this questionnaire (*r* = 0.365, paired observations *n* = 277). Interitem reliability was acceptable (α = 0.76–0.84).

Neighborhood safety was assessed with a 5-item observational measure completed by a trained data collector who rated the safety of the child’s neighborhood. Five items assessing safety adapted from PhenX Toolkit “Neighborhood Safety” (Mujahid et al., 2007) were answered by the data collector on a 5-point Likert scale, ranging from 1 (“strongly disagree”) to 5 (“strongly agree”). Sample items include “this is a safe neighborhood for children to play unattended” and “this is a safe neighborhood for someone to walk alone in the evening.” Item scores were aggregated to create a composite score (α = 0.90).

##### Macrosystem-level predictors

Positive societal and cultural predictors at the macrosystem level included religious/spiritual affiliation and financial security. Parents self-reported the importance of religious and spiritual affiliations in everyday life on a 4-point Likert scale ranging from 1 (“very important”) to 4 (“not at all important”) (Conger et al., 1994). A mean score of the two parents’ ratings was used when two parents completed the questionnaire (*r* = 0.678, paired observations *n* = 366).

Household financial security was assessed using the making ends meet subscale of the Family Financial Questionnaire (Conger et al., 1992, 1994). Each parent reported whether they have difficulty paying bills each month or have trouble making ends meet on a 5-point Likert scale, ranging from 1 (“great difficulty”) to 5 (“no difficulty”). In this study, the subscale was reverse coded so that higher scores indicated greater financial stability (i.e., parent reported no difficulty paying bills or making ends meet each month). When two parental ratings were present, a mean score of the two parents’ ratings was used (*r* = 0.624, paired observations *n* = 339). Interitem reliability was acceptable (α = 0.69–0.72).

#### Risk factors: biological family psychopathology and prenatal substance use

Two bioecological risk factors were included as predictors, using constructs derived in prior EGDS studies (Kerr et al., 2013; Natsuaki et al., 2013; Neiderhiser et al., 2016). Risks associated with biological family psychopathology, specifically internalizing behavior problems and externalizing behavior problems, were indexed by composite measures of birth parent and birth family characteristics, including (a) birth parent psychopathology symptoms, (b) birth parent diagnoses, (c) birth parent age of onset of disorders, and (d) proportion of birth parent’s first-degree relatives (mother, father, and up to three siblings) experiencing problems with psychopathology. These composites were created using principal component analyses based on the four aforementioned components. Internalizing problems included agoraphobia, agoraphobia without panic, adult separation anxiety, dysthymia, generalized anxiety disorder, major depression, panic disorder, recurrent brief depression, separation anxiety, and social phobia assessed with the Composite International Diagnostic Interview (CIDI; Kessler and Üstün, 2004). Externalizing problems included conduct disorder and antisocial personality assessed with the Diagnostic Interview Schedule (DIS; Robins, 1981). The composites have been used previously with this EGDS sample in multiple reports (e.g., Marceau et al., 2019; Liu et al., 2020). See Marceau et al. (2019) for more details on the rationale for the score creation.

Prenatal substance use was operationalized as a binary variable (yes/no) using a perinatal risk index as developed and described thoroughly by Marceau et al. (2016). Data on substance use during pregnancy were collected from birth mothers at 3–6 months post-partum. The comprehensive coding system, based on the McNeil-Sjostrom Obstetric Complications Scale (McNeil et al., 1994) and a previous systematic review on prenatal toxin exposure (Williams and Ross, 2007), included reported use of cigarettes, alcohol, marijuana, cocaine, hallucinogens, amphetamines, heroin, prescription painkillers (used illegally), inhalants, sedatives, tranquilizers and/or being exposed to second hand smoke. Coded scores that reflected prenatal substance use that may pose risk to the fetus were assigned a one (yes); if the mother’s total score did not reflect substance use that may pose a risk to the fetus, they were assigned a zero (no).

#### Covariates

We accounted for sex assigned at birth, child race, child ethnicity, and age 7 prosocial behavior in the analyses. Sex assigned at birth was included given previously identified sex and gender differences in prosocial tendencies (Eagly and Koenig, 2006). Age 7 prosocial behavior was included to control for earlier levels of prosocial behavior. It was measured using the social skills subscale of the Social Skills Rating System (SSRS; Gresham and Elliott, 1990), because the SDQ, which measured outcome prosocial behavior at age 11, was not collected earlier in the study. The SSRS social skills measure was significantly correlated with the SDQ social skills measure (*r* = 0.218, *p* < 0.001). The SSRS total social skills scale is a composite of 10-item measures of cooperation (helping out), assertion (starting conversations, making friends easily), responsibility (asking permission, reporting accidents), and self-control (controls temper, responds appropriately when hit or pushed by other children). Parents rated each item on a scale of 0 (“never”) to 2 (“very often”). When there were two parents’ reports, a mean score of the two parents’ ratings was used (*r* = 0.49). Interitem reliability was acceptable (α = 0.87–0.92).

#### Moderator

Although considered a covariate when testing Hypothesis 1 and 2, household type was additionally included as a moderator when testing Hypothesis 3. Household type reflects the two types of households in the current study: adoptive families in which an adopted child was raised from early infancy by non-relative parent (s) or biological families in which the adoptees’ biological sibling were raised by their biological parent (s). Household type was coded as 0 (“adoptive household”) and 1 (“biological household”). In the current study, the adopted child and biological sibling are not matched pairs, and are analyzed by their group (i.e., adoptive household or biological household) rather than by their sibling relationship. This decision was made due to the reduction in sample size and associated reduction in power, if a requirement for pair-ship was imposed on the data.

### Analytic procedures

To test the study aims, we used multiple regression to examine the effects of potential promotive and protective factors on prosocial behaviors at age 11 while also considering biological family psychopathology and prenatal substance use. First, we estimated a linear regression model to test whether positive factors across bioecological levels predicted prosocial behavior at age 11 when controlling for household type and the aforementioned covariates. Then, we added the risk factors to the model to examine which promotive factors remain significant when also controlling for the risk factors (Research Question 1). Next, we added multiplicative interaction terms between the risk factors and any significant promotive factors from the main effects model, to test whether it had a protective effect (i.e., moderated) on the relationships between one of the risk factors and age 11 prosocial behavior (Research Question 2).

Third, we added household type to the interaction terms to examine whether protective effects may vary by home environment type (Research Question 3). For both research questions 2 and 3, any significant interactions were decomposed by estimating simple intercepts and slopes to determine the region of significance, and marginal effects were estimated to graph the interactions. Fourth, we ran unadjusted bivariate comparisons between all predictor variables and household type to further understand differences between them and contextualize the findings (Research Question 4). To assess the magnitude of these differences, we used independent *t*-tests and Cohen’s *d* effect sizes for continuous variables or chi-square tests of independence and odds ratio effect sizes for categorical variables.

#### Data analysis

We first explored the data by calculating descriptive statistics, running bivariate correlations, and identifying missingness patterns. There was a moderate amount of missingness in the independent variables (2–30%). Missing data were handled through multiple imputation using chained equations (White et al., 2011) in which results from the 25 imputed datasets were pooled in accordance with Rubin’s rules (Rubin, 1987). Due to data not being missing completely at random [Little’s MCAR test χ^2^ (258) = 730.23, *p* < 0.001; Little, 1988], *post-hoc* sensitivity tests were conducted to account for complete vs. incomplete data in the imputation models; findings did not differ, and we proceeded with using the original imputed data.

Regression diagnostics were run to assess whether the assumptions of normality, homoscedasticity, and linearity were met and identify if multicollinearity or influential cases were present. All linear regression assumptions were met other than that of homoscedasticity. As such, all models were estimated using cluster robust standard errors to address heteroscedasticity and account for a small amount of clustering within families (i.e., when an adopted child shared a birth parent (s) with a biological child in the study; ICC = 0.001, design effect = 1.00). All analyses assessed statistical significance at the α = 0.05 level.

Statistical analyses were performed in R (v4.1.3; R Core Team, 2022) with the following packages: mice (v3.15.0; van Buuren and Groothuis-Oudshoorn, 2011), miceadds (v3.16–18; Robitzsch et al., 2017), estimatr (v1.0.0; Blair et al., 2021), and ggeffects (v1.3.1; Lüdecke, 2018). Simple slopes and the region of significance were identified using the online calculator from Preacher et al. (2023).

## Results

### Analytic sample characteristics

Descriptive statistics and bivariate correlations for the study variables included in the analyses are presented in Tables 2, 3, respectively. Measures of continuous variables’ symmetry are provided in Supplementary Table S1.

**Table 2:** Descriptive statistics and comparisons of participant characteristics across household types.

		Adoptive Household *n* = 386 (83%)		Biological Household *n* = 80 (17%)		Adopted vs. Biological Child	
Variables	Range	*M* or %	*SD*	*M* or %	*SD*	*d*	*OR*
Child Sex							
Male	0–1	57.77	%	42.50	%	—	1.85*
Female	0–1	42.23	%	57.50	%	—	0.54*
Child Race & Ethnicity							
White non-Hispanic	0–1	55.44	%	62.50	%	—	0.75
Multiple races	0–1	24.87	%	13.75	%	—	2.08*
Black	0–1	12.44	%	16.25	%	—	0.73
Other race or ethnicity	0–1	7.25	%	7.50	%	—	0.96
Prenatal substance use	0–1						
No	0–1	60.62	%	70.20	%	—	0.64
Yes	0–1	39.38	%	29.80	%	—	1.53
BF externalizing behaviors	−3.7–4.8	0.03	1.80	0.38	1.73	0.01	—
BF internalizing behaviors	−3.5–5.4	0.02	1.56	0.04	1.65	0.20	—
Prosocial baseline	10.0–72.5	49.82	8.63	45.41	12.22	−0.47**	—
Prosocial age 11	1.0–10.0	8.31	1.59	7.35	2.05	−0.57**	—
Positive affectivity	3.4–7.0	5.86	0.53	5.79	0.74	−0.12	—
Parental warmth	27.5–42.0	37.86	3.27	38.72	3.46	0.26	—
Neighborhood safety	2.0–4.80	4.56	0.31	3.90	0.62	−1.73**	—
Neighborhood involvement	1.0–3.0	1.94	0.50	1.48	0.49	−0.92**	—
Importance of religion	1.0–4.0	1.98	0.94	2.31	1.01	0.35**	—
Financial security	1.0–8.0	6.27	1.39	4.91	1.66	−0.94**	—

*Notes*. Results are pooled estimates across 25 multiply imputed datasets. Prosocial behavior at age 7 was collected with the SSRS and prosocial behavior at age 11 was collected with the SDQ. BF = biological family; *M* = mean; *SD* = standard deviation; *d* = standardized mean difference effect size; *OR* = odds ratio effect size; *p <0.05, **p <0.01.

**Table 3:** Bivariate correlations between independent and dependent variables.

Variable	1	2	3	4	5	6	7	8	9	10	11	12	13	14	15	16
Child Sex	―															
Prosocial (11)	0.071	―														
BF externalizing	−0.011	−0.065	―													
BF internalizing	−0.034	−0.145**	0.254**	―												
Prenatal substance use	0.018	−0.044	0.224**	0.173**	―											
Prosocial (7)	0.131*	0.218**	−0.089	−0.088	−0.044	―										
Positive affectivity	0.162**	0.207**	0.000	0.032	−0.062	0.386**	―									
Parental warmth	0.080	0.162**	0.019	0.053	−0.093	0.222**	0.289**	―								
Neighborhood involvement	−0.023	0.146**	−0.098	0.103	0.000	0.085	0.188	−0.065	―							
Religion importance	0.066	−0.019	0.000	−0.001	0.016	−0.080	−0.045	0.034	−0.023	―						
Financial stress	−0.027	0.063	−0.116*	0.055	−0.010	0.157*	−0.004	0.005	0.175	0.022	―					
Household type	−0.116*	0.212**	−0.073	−0.004	0.075	0.176**	0.048	−0.099*	0.331	−0.133**	0.335**	―				
Neighborhood safety	−0.098*	0.141**	−0.062	−0.021	0.001	0.112*	0.066	−0.033	0.149	−0.033	0.238**	0.549**	―			
White non-Hispanic	−0.004	−0.028	0.066	0.037	0.111*	−0.078	0.030	−0.027	0.067	0.075	0.057	−0.054	0.056	―		
Multiple races	0.062	0.090	0.021	0.102*	−0.010	0.176**	0.065	0.080	0.022	−0.007	0.023	0.100*	0.035	−0.624**	―	
Other race or ethnicity	0.046	0.043	0.033	−0.106*	0.026	0.029	−0.005	0.031	−0.070	−0.014	−0.042	−0.004	0.002	−0.321**	−0.153*	―
Black	−0.107*	−0.105*	−0.148**	−0.100*	−0.171**	−0.127*	−0.120*	−0.084	−0.072	−0.091	−0.080	−0.043	−0.128**	−0.444**	−0.212**	−0.109**

*Notes*. Results are pooled estimates across 25 multiply imputed datasets. Reference group: Male for child sex, where 0 = male, 1 = female; Biological household for household type, where 0 = biological household, 1 = adoptive household; BF = biological family; *p < 0.050, **p < 0.010.

### Research question 1

The results of the full main effects model are shown in the first panel of Table 3, labeled Model 1. Among the positive bioecological factors examined, only parental warmth had a significant promotive effect on prosocial behavior (*b* = 0.06, *p* = 0.028, 95% CI [0.01, 0.12]). Household type was also a significant predictor of prosocial behavior, with youth living in adoptive homes reporting significantly higher prosocial behavior than youth living in biological homes (*b* = 0.80, *p* = 0.009, 95% CI [0.20, 1.40]). The results of the main effects after controlling for risk factors are shown in the second panel of Table 3, labeled Model 2. Biological family internalizing behavior was significantly associated with prosocial behavior, with higher biological family internalizing behavior predicting lower child prosocial behavior (*b* = −0.17, *p* = 0.008, 95% CI [−0.30, −0.04]). Parental warmth remained a significant predictor of prosocial behavior (*b* = 0.07, *p* = 0.016, 95% CI [0.01, 0.13]), as did household type (*b* = 0.80, *p* = 0.010, 95% CI [0.19, 1.40]).

### Research question 2

Because parental warmth had a robust promotive effect on prosocial behavior, we next tested whether it had protective effects on the relationships between the identified risk associated with biological family psychopathology and prenatal substance use with child prosocial behavior. We did not find evidence of this, as none of the 2-way interactions were significant, with all estimates having *p* ≥ 0.050 (Supplementary Table S2).

### Research question 3

**Figure 2:**
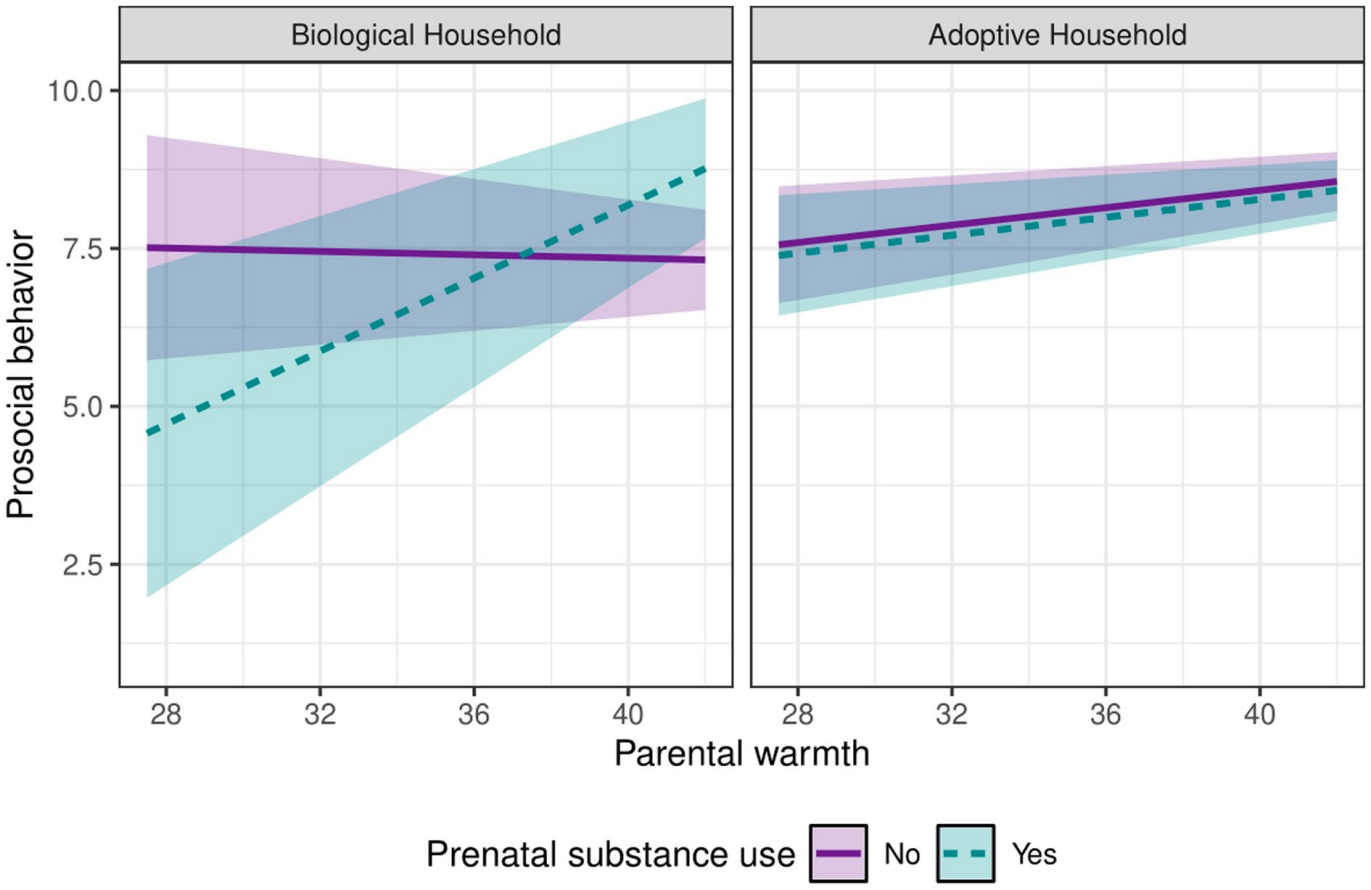
Interaction between prenatal substance use and parental warmth on prosocial behavior at age 11 across household types. Parameters were adjusted for child sex, race, ethnicity, and age 7 prosocial behavior covariates.

Next, we examined whether a potential protective effect of parental warmth on prosocial behavior differed between household type, which represented different rearing environments—one that is free from the influence of intergenerational genetic transmission within the rearing environment (adoptive parent home) and one that included the influence of intergenerational genetic transmission in the rearing environment (biological parent home). Analyses indicated that the effect of parental warmth on the relationship between prenatal substance use and child prosocial behavior significantly differed by household type, *b* = −0.30, *p* = 0.025, 95% CI [−0.56, −0.04] (Table 4). Decomposition of the conditional regression of prosocial behavior on prenatal substance use as a function of parental warmth and household type revealed that the simple slopes for biological households were significant at values −2.45 SD below and +1.04 SD above mean parental warmth, but there was no region of significance for adoptive households. Plotting these conditional effects (Figure 2) revealed that parental warmth had a protective effect on the relationship between prenatal substance use and children’s prosocial behavior in biological households, such that children exposed to prenatal substance use and raised with biological parents who reported higher levels of warmth toward their child (+1.04 SD) had higher prosocial behaviors. For adopted children, parental warmth did not impact the association between prenatal drug use and prosocial behavior. In all other models, the potential protective effect of parental warmth on risks associated with biological family externalizing behavior problems and internalizing behavior problems did not vary across household type (Supplementary Table S3).

**Table 4:** Main effects predicting prosocial behavior at age 11.

	Model 1			Model 2		
Variables	*b*	*SE*	95% CI	*b*	*SE*	95% CI
***Covariates***						
Child sex	0.16	0.15	−0.14, 0.47	0.14	0.15	−0.16, 0.44
Multiple races	0.13	0.18	−0.22, 0.48	0.18	0.18	−0.17, 0.54
Other race or ethnicity	0.26	0.27	−0.27, 0.79	0.16	0.27	−0.36, 0.69
Black	−0.21	0.26	−0.72, 0.31	−0.31	0.27	−0.84, 0.22
Household type	0.80**	0.31	0.20, 1.40	0.80*	0.31	0.19, 1.40
Prosocial age 7	0.02	0.01	−0.01, 0.04	0.01	0.01	−0.01, 0.04
***Individual***						
Positive affectivity	0.28	0.20	−0.12, 0.68	0.30	0.20	−0.09, 0.69
***Family***						
Parental warmth	0.06*	0.03	0.01, 0.12	0.07*	0.03	0.01, 0.13
***Community***						
Neighborhood involvement	0.24	0.18	−0.12, 0.59	0.28	0.18	−0.07, 0.63
Neighborhood safety	0.10	0.23	−0.35, 0.55	0.07	0.23	−0.38, 0.52
***Macro-level***						
Financial security	−0.04	0.06	−0.15, 0.08	−0.03	0.06	−0.14, 0.09
Importance of religion	0.02	0.09	−0.16, 0.19	0.01	0.09	−0.16, 0.18
***Risk factors***						
BF externalizing behaviors				−0.01	0.05	−0.11, 0.10
BF internalizing behaviors				−0.17**	0.06	−0.30, −0.04
Prenatal substance use				−0.06	0.17	−0.39, 0.26

*Notes.* Results are pooled estimates across 25 multiply imputed datasets; Reference groups: Male for child sex, where 0 = male, and 1 = female; White Non-Hispanic for race & ethnicities; and biological household for household type, where 0 = biological household, 1 = adoptive household.

BF = biological family; *b* = unstandardized beta coefficient; SE = cluster robust standard error; 95% CI = 95% confidence interval around unstandardized beta coefficient. *p <0.05, **p <0.01.

### Research question 4

Bivariate comparisons between the adopted children (*n* = 386, 83%) and biological children (*n* = 80, 17%) are presented in Table 2, in the final column. Analyses indicated that biological households had significantly lower levels of neighborhood involvement (*d* = −0.92, *p* < 0.001, financial security*, d* = −0.94, *p* < 0.001), and neighborhood safety (*d* = −1.73, *p* < 0.001) compared to adoptive households in this study. Biological households had significantly higher reported importance of religious beliefs in their everyday life, compared to adoptive households (*d* = 0.35, *p* = 0.005) Children raised in biological families also had lower levels of prosocial behavior at both age 7 (*d* = −0.47, p < 0.001) and age 11 (*d* = −0.57, *p* < 0.001) than children raised adoptive families. The lower neighborhood safety and financial insecurity relative to the adoptive households suggest that biological households may face greater adversity, especially in the ecosystem and macrosystem levels. There were no significant differences between the households in child smiling and laughing, parental warmth, prenatal substance use, and biological family psychopathology (internalizing behavior problems and externalizing behavior problems).

## Discussion

We sought to examine promotive, and potentially protective, associations between multiple aspects of the bioecological system at age 7 and children’s prosocial behavior at age 11. The results highlight the importance of children’s microsystem—in particular, parental warmth—on children’s prosocial behavior development. We tested four hypotheses, some of which were partially supported. Hypothesis 1, that all bioecological factors would have a significant association with later prosocial behavior, was only partially supported. Specifically, parental warmth at age 7, a microsystem level factor, was the only bioecological predictor that was promotive of child prosocial behavior. The observed longitudinal associations between parental warmth and later prosocial behavior are consistent with extant literature (Grusec and Goodnow, 1994; Eisenberg and Fabes, 1998; Knafo and Plomin, 2006; Padilla-Walker and Christensen, 2011), emphasizing the importance of family and parental relationships in child prosocial development. The current study adds to the prior body of work by demonstrating the unique variance in age 11 prosocial behavior accounted for by parental warmth, even when several risk factors and a range of other promotive factors in the bioecological system were considered in the model.

In the second hypothesis, we expected a protective effect from parental warmth in the context of both biological family psychopathology and/or prenatal substance use. Results did not support this hypothesis. Parental warmth did not buffer the association between biological family psychopathology (internalizing or externalizing problems) or prenatal substance use on age 11 prosocial behavior within the full sample. However, as indicated when we tested our third hypothesis, there was an indication that parental warmth did moderate the effects of prenatal substance in the biological households.

That leads us to our third hypothesis where we aimed to examine the predictive models testing in Hypothesis one and two by household type, to see if some patterns were evidence in one household type but not the other. As expected, the associations between parental warmth, prenatal substance use, and age 11 prosocial behavior varied by household type. In contrast, household type did not significantly moderate the association between parental warmth, biological family psychopathology, and age 11 prosocial behavior. The significant three-way interaction with prenatal substance use showed that, in context of prenatal substance use, parental warmth had a protective association with child prosocial behavior in households when children were raised by their biological parents. Conversely, this association was not found when children were raised by adoptive parents. Results from our exploratory Research Question 4 revealed that there were no significant differences in rates of prenatal drug exposure or in levels of parental warmth between household type, suggesting that the moderated association found in the current study was not the result of between group mean-level differences in these specific risk or protective factors.

Taken together, these findings highlight the potential for parental warmth to be both an asset in all families (as evidenced by its main effect in all models), as well as its function as a resilience factor in face of exposure to prenatal substance use, when children remain with their biological parent (s). In biological homes where there has been prenatal exposure to maternal substance use, parental warmth may enable adaptation in context of adversity. Conversely, within biological homes, children self-reported fewer prosocial tendencies if their biological mother reported prenatal substance use and she also reported lower levels of parental warmth.

The association between parental warmth and prosocial behavior among children in biological families may reflect intergenerational genetic transmission processes due to the fact that in the biological homes, the parent and child shared common genes. Specifically, parents and their biological children share 50% of their genetic make-up, and the same genes that influence parental warmth may influence prosocial behavior (Scarr and McCartney, 1983). However, the persistent main effect of parental warmth on child prosocial across all models and family configurations suggests that its role is also environmental. Being able to examine these associations within the context of shared genes (i.e., in biological families) as well as in families where children and their rearing parents do not share genes adds to the novelty of the current study and highlights with role of parental warmth as an environmental mechanism of transmission of prosocial behavior.

Differences between the household types themselves, such as resources or characteristics of the environment, may have also contributed to the strength of the associations found in the current study. To further contextualize possible operating mechanisms behind the moderation results in the current study, our fourth hypothesis explored potential differences in the bioecological factors and risk factors between the two household types. Such analyses can provide insights into whether the operating mechanism in the moderation effect for household type may be due to qualities of the two household types that differ, rather than anything specific to household type itself (e.g., that the child is adopted and does not share genes with their rearing parent). In the current study, these analyses indicated that children in biological households had lower resources compared to adoptive households. Specifically, biological households had lower neighborhood safety and greater financial insecurity, indicating different exosystem and macrosystem environments between the two household types. The differences in resources in rearing environments may partially explain the moderating effect of parental warmth on prenatal substance use found only in the biological households. For example, the unique protective effect in biological households of parental warmth against prenatal substance on prosocial behavior may result from shared genes between the biological mother and child they are parenting, and/or, it may be that experiencing high parental warmth in context of less financial security and less involvement in the community can bolster a child’s ability to develop prosocial behavior. With either rationale, parental warmth is an important influence on the development of early adolescent prosocial behavior. It is promotive across households for prosocial behavior, regardless of environment, and protective in biological households—which are marked by lower financial security and less involvement in the community when there is history of prenatal substance use. Separating out the role of financial security and neighborhood involvement from the role of household type was not an aim of the current study, but our findings indicated it is an important future direction. Implications could extend to providing parenting and family-support interventions to support child prosocial behavior with parents who have used substances prenatally and are lower-resourced. This finding also acknowledges the bioecological nature of prosocial behavior development, such that numerous levels interact to promote its development. As such, interventions to encourage prosocial behavior ought to engage factors across multiple bioecological levels.

### The benefits of parental warmth: connections to the intervention literature

In the current analytic sample, the protective effect of parental warmth was particularly important for biological parent–child households who had a history of substance use during pregnancy. These findings provide evidence to support interventions focused on fostering warm, attentive parenting styles with mothers who used substances. Attachment-based parenting interventions, such as the Mothers and Toddlers Program (Suchman et al., 2011) have been shown to be effective at improving mother–child warmth and have been associated with positive sequalae that arise from parental warmth (Suchman et al., 2011). These interventions are supported by the link between warm and sensitive parenting, related secure attachment of mother and child, and child prosocial behavior (Eisenberg et al., 2006). A systematic review of integrated programs for mothers with substance use found that interventions that included components to develop parent–child relationships and attachment by way of parental warmth, resulted in both positive treatment effects for mothers and beneficial effects for children (Niccols et al., 2012). Other researchers and practitioners point to importance of focusing on family strengthening interventions that focus on warm parenting particularly in risky contexts to foster positive prosocial development. For example, for families experiencing material hardship, positive parenting (including warm, responsive parenting) mediated the negative effects of material hardship on prosocial behavior (Lee et al., 2021). In addition, numerous studies have shown that parenting interventions are often more effective for lower-resourced families when compared to higher-resourced families (Shaw et al., 2016; Pelham et al., 2017).

### Study limitations and future directions

There are several limitations to consider when interpreting and applying the current results. There was missing data, which addressed with imputation to allow for a more complete analytic sample. However, some measures we had originally hoped to include in the analyses were not considered as they were not suitable for imputation. Specifically, inclusion of positive student-teacher relationships would have made an additional contribution to the model by adding another variable in the exosystem. However, there was a high amount of missingness in teacher-reported variables as the response rates were less than 50%, and as such, we did not include this variable in our models. Consequently, the analysis included only a subset of theoretically possible risk and promotive factors that may be associated with the development of prosocial behavior.

In addition, the current analysis was limited by a relatively culturally homogenous sample and few measures of cultural practices and norms. Future directions include exploring the role of multiculturalism in prosocial development since social responsibility and altruistic tendencies vary across cultures (Carlo and Padilla-Walker, 2020). Relatedly, the associations of certain factors may change with a more diverse sample in terms of socioeconomic status, race, ethnicity, education level, and other important factors that allow for more comprehensive representativeness. While including both adoptive and biological families increased heterogeneity, the biological families made up less than a quarter of the analytical sample. A greater range of perceived financial security, particularly households with less security and more financial stress, may have changed the results. Thus, the current study is an illuminating but incomplete picture of the child and family’s experiences.

Although a strength of the study was incorporating numerous contextual factors from different ecological levels, the types of measures posed limitations. To start, this study used biological family internalizing and externalizing behaviors as an indicator of family risk for psychopathology. However, we did not specifically hypothesize or test for gene–environment interaction, given the analyses included both adoptive and biological parent–child dyads so the tests of environmental moderation do not directly illuminate gene–environment interaction in biological families. As the parent study’s initial data collection did not include indicators of moral development, our study’s scope was limited to biological and environmental influences on prosocial behavior. Future studies can explore how moral development can provide an additional theoretical lens through which to consider how factors across various ecological levels can influence the development of prosocial behavior. Another limitation of our measurement was that both baseline prosocial behavior and the outcome prosocial behavior were measured as prosocial behavior toward others, in general. Prosocial behavior can be specific to certain contexts, such as within families or toward parents, peers, or strangers. Subsequent studies can further differentiate the recipient of prosocial behavior and the context in which it is delivered.

There was potential for bias in all self-reported measures (e.g., early adolescent self-reported prosocial behavior or parent self-reported parental warmth) Future research can use observations or alternative reports to improve objectivity and decrease the risk of bias. Additionally, neighborhood safety was measured by a trained observer who reported on elements of apparent safety and signs of delinquent behavior, which has the risk of implicit bias. We aimed to select questions that had the least potential for bias, but there was still the possibility that biased heuristics were used when rating neighborhood quality (O’Brien and Kauffman, 2013).

Although our findings that show that parental warmth can buffer risk from prenatal substance use and promote prosocial behavior in biological families are congruent with existing research, the reduced statistical power of our models given the sample size in the biological households is also a limitation. Replicated analysis with additional power are needed to elaborate on the protective effects of parental warmth across rearing environments.

This study contributes to the field of child development by examining key bioecological factors in a child’s environment that can affect their prosocial development. The results emphasize that a child’s social context, particularly warm relationships with their parents, shape their prosocial development. The benefit of warm parenting in buffering risk from prenatal substance use in low-resourced households (i.e., biological families in the current study) identified parental warmth as a key part of a child’s and family’s resilience process. Future research can extend these findings and assess the moderating role of resource availability on the parental warmth-prosocial behavior association. In addition, subsequent work can examine the longitudinal association of prosocial behavior and parental warmth in context of environmental risk factors, as the current study looked only at prenatal substance use and biologic family psychopathology risk factors. A strength-based approach to different adverse contexts can explore what resources families possess and can use to overcome challenges and promote their child’s development.

The current findings hold important implications for practice. They can be used in support of parenting interventions and broader interventions to support parents in promoting a child’s prosocial behavior. In particular, interventions that promote parental warmth, especially for parents who have used substances, can be beneficial for the child’s prosocial development. Moreover, there are potential policy implications. Prior research suggests that punitive policies toward prenatal substance use are associated with adverse pregnancy outcomes, whereas supportive policies (i.e., connections with substance use treatment) are more effective for enhancing prenatal health (Meinhofer et al., 2022). In a sense, supportive policies are inherently prosocial, and may possibly foster more prosocial behaviors in the families who benefit from them. Further, linking practice and policy, such as integrating parenting interventions into supportive prenatal substance use policies may provide an early intervention route. Taken together, this study supports the impact of parenting on a child’s prosocial development and serves as a stepping stone to guide future work into how to bolster child and family strengths to promote prosocial development and resilience.

## Data availability statement

Data are not publicly available due to privacy or ethical restrictions, but are available on request from the corresponding author (EB, ebates@uoregon.edu).

## Ethics statement

The studies involving human participants were reviewed and approved by Institutional Review Boards at the University of Oregon and the Pennsylvania State University. The studies were conducted in accordance with the local legislation and institutional requirements. The adult participants and minors’ legal guardians provided their written informed consent for participation in this study. In addition, youth age 7 and older provided their assent to participate in this study.

## Author contributions

EB: Conceptualization, Formal analysis, Methodology, Writing – original draft, Writing – review & editing. LB: Conceptualization, Formal analysis, Methodology, Software, Visualization, Writing – review & editing. JG: Funding acquisition, Investigation, Writing – review & editing. MN: Investigation, Writing – review & editing. JN: Funding acquisition, Investigation, Writing – review & editing. DS: Investigation, Writing – review & editing. LL: Conceptualization, Funding acquisition, Investigation, Methodology, Supervision, Writing – review & editing.

## References

[ref1] Almedom A. M. (2005). Social capital and mental health: an interdisciplinary review of primary evidence. Soc. Sci. Med. 61, 943–964. doi: 10.1016/j.soscimed.2004.12.025, PMID: 15955397

[ref2] Bada H. S. Bann C. M. Whitaker T. M. Bauer C. R. Shankaran S. LaGasse L.. (2012). Protective factors can mitigate behavior problems after prenatal cocaine and other drug exposures. Pediatrics 130, e1479–e1488. doi: 10.1542/peds.2011-3306, PMID: 23184114 PMC3507246

[ref3] Bandura A. (1977). Social learning theory. Englewood Cliffs, NJ: Prentice Hall.

[ref9001] Biglan A. Hinds E. (2009). Evolving prosocial and sustainable neighborhoods and communities. Annu. Rev. Clin. Psychol. 5, 169–196. doi: 10.1146/annurev.clinpsy.032408.153526 19327029 PMC2663939

[ref4] Blair G. Cooper J. Coppock A. Humphreys M. Sonnet L. Fultz N. (2021). Estimatr: fast estimators for design-based inference. R package version 1.0.0 Available at: https://CRAN.R-project.org/package=estimatr

[ref5] Borowski S. K. Groh A. M. Bakermans-Kranenburg M. J. Fearon P. Roisman G. I. Van IJzendoorn M. H.. (2021). The significance of early temperamental reactivity for children’s social competence with peers: a meta-analytic review and comparison with the role of early attachment. Psychol. Bull. 147, 1125–1158. doi: 10.1037/bul0000346, PMID: 35238583

[ref6] Boyce W. T. Levitt P. Martinez F. D. McEwen B. S. Shonkoff J. P. (2021). Genes, environments, and time: the biology of adversity and resilience. Pediatrics 147:e20201651. doi: 10.1542/peds.2020-1651, PMID: 33495368

[ref7] Bronfenbrenner U. (1974). Developmental research, public policy, and the ecology of childhood. Child Dev. 45:1. doi: 10.2307/1127743

[ref8] Bronfenbrenner U. Morris P. A. (2007). “The bioecological model of human development” in Handbook of child psychology. eds. Damon W. Lerner R. M. (Hoboken, NJ: John Wiley & Sons, Inc)

[ref9] Buuren S. V. Groothuis-Oudshoorn K. (2011). Mice: multivariate imputation by chained equations in R. J. Stat. Soft. 45, 1–67. doi: 10.18637/jss.v045.i03

[ref10] Carlo G. Padilla-Walker L. (2020). Adolescents’ prosocial behaviors through a multidimensional and multicultural Lens. Child Dev. Perspect. 14, 265–272. doi: 10.1111/cdep.12391

[ref11] Carlo G. White R. M. B. Streit C. Knight G. P. Zeiders K. H. (2018). Longitudinal relations among parenting styles, prosocial behaviors, and academic outcomes in U.S. Mexican Adolesc. Child Dev. 89, 577–592. doi: 10.1111/cdev.12761, PMID: 28213904 PMC5562534

[ref12] Chetty R. Hendren N. (2018). The impacts of neighborhoods on intergenerational mobility I: childhood exposure effects. Q. J. Econ. 133, 1107–1162. doi: 10.1093/qje/qjy007

[ref13] Chyn E. Katz L. F. (2021). Neighborhoods matter: assessing the evidence for place effects. J. Econ. Perspect. 35, 197–222. doi: 10.1257/jep.35.4.197

[ref14] Conger R. D. Conger K. J. Elder G. H. Lorenz F. O. Simons R. L. Whitbeck L. B. (1992). A family process model of economic hardship and adjustment of early adolescent Boys. Child Dev. 63, 526–541. doi: 10.2307/1131344, PMID: 1600820

[ref15] Conger R. D. Ge X. Elder G. H. Lorenz F. O. Simons R. L. (1994). Economic stress, coercive family process, and developmental problems of adolescents. Child Dev. 65:541. doi: 10.2307/1131401 8013239

[ref16] Coussons-Read M. E. (2013). Effects of prenatal stress on pregnancy and human development: mechanisms and pathways. Obst. Med. 6, 52–57. doi: 10.1177/1753495x12473751, PMID: 27757157 PMC5052760

[ref17] Crnic K. A. Greenberg M. T. Ragozin A. Z. Robinson M. N. Basham R. (1983). Effects of stress and social support on mothers and premature and full-term infants. Child Dev. 54, 209–217. doi: 10.2307/1129878 6831987

[ref18] Davis M. Suveg C. (2014). Focusing on the positive: a review of the role of child positive affect in developmental psychopathology. Clin. Child. Fam. Psychol. Rev. 17, 97–124. doi: 10.1007/s10567-013-0162-y 24323039

[ref19] de Water E. Rockhold M. N. Roediger D. J. Krueger A. M. Mueller B. A. Boys C. J.. (2021). Social behaviors and gray matter volumes of brain areas supporting social cognition in children and adolescents with prenatal alcohol exposure. Brain Res. 1761:147388. doi: 10.1016/j.brainres.2021.147388, PMID: 33621483 PMC8377082

[ref20] Dunn E. C. Soare T. W. Zhu Y. Simpkin A. J. Suderman M. J. Klengel T.. (2019). Sensitive periods for the effect of childhood adversity on DNA methylation: results from a prospective, longitudinal study. Biol. Psychiatry 85, 838–849. doi: 10.1016/j.biopsych.2018.12.023, PMID: 30905381 PMC6552666

[ref21] Eagly A. H. Koenig A. M. (2006). “Social role theory of sex differences and similarities: implication for prosocial behavior” in Sex differences and similarities in communication. eds. Dindia K. Canary D. J. (Mahwah, NJ: Lawrence Erlbaum Associates Publishers), 161–177.

[ref22] Eisenberg N. Fabes R. A. (1998). “Prosocial development” in Handbook of child psychology: Social, emotional, and personality development. eds. Damon W. Eisenberg N. (Hoboken, NJ: John Wiley & Sons, Inc), 701–778.

[ref23] Eisenberg N. Fabes R. A. Spinrad T. L. (2006). “Prosocial development” in Handbook of child psychology: Social, emotional, and personality development. eds. Eisenberg N. Damon W. Lerner R. (Hoboken, NJ: John Wiley & Sons, Inc), 646–718.

[ref24] Eisenberg N. VanSchyndel S. K. Spinrad T. L. (2016). Prosocial motivation: inferences from an opaque body of work. Child Dev. 87, 1668–1678. doi: 10.1111/cdev.12638, PMID: 28262941

[ref25] Eisenberg N. Vaughan J. Hofer C. (2009). “Temperament, self-regulation, and peer social competence” in Handbook of peer interactions, relationships, and groups. eds. Rubin K. H. Bukowski W. Laursen B. (New York, NY: The Guilford Press), 473–489.

[ref26] Flook L. Zahn-Waxler C. Davidson R. J. (2019). Developmental differences in prosocial behavior between preschool and late elementary school. Front. Psychol. 10:876. doi: 10.3389/fpsyg.2019.00876, PMID: 31080421 PMC6497732

[ref27] Flouri E. Sarmadi Z. (2016). Prosocial behavior and childhood trajectories of internalizing and externalizing problems: the role of neighborhood and school contexts. Dev. Psychol. 52, 253–258. doi: 10.1037/dev0000076, PMID: 26619321 PMC4725335

[ref28] Fowler J. H. Christakis N. A. (2010). Cooperative behavior cascades in human social networks. Proc. Natl. Acad. Sci. U. S. A. 107, 5334–5338. doi: 10.1073/pnas.0913149107, PMID: 20212120 PMC2851803

[ref29] Furrow J. L. King P. E. White K. (2004). Religion and positive youth development: identity, meaning, and prosocial concerns. Appl. Dev. Sci. 8, 17–26. doi: 10.1207/S1532480XADS0801_3

[ref30] Gerell M. (2015). Collective efficacy, neighborhood and geographical units of analysis: findings from a case study of Swedish residential neighborhoods. Eur. J. Crim. Policy Res. 21, 385–406. doi: 10.1007/s10610-014-9257-3

[ref31] Goodman R. (1997). The strengths and difficulties questionnaire: a research note. J. Child Psychol. Psychiatry 38, 581–586. doi: 10.1111/j.1469-7610.1997.tb01545.x 9255702

[ref32] Goodman R. (2001). Psychometric properties of the strengths and difficulties questionnaire. J. Am. Acad. Child Adolesc. Psychiatry 40, 1337–1345. doi: 10.1097/00004583-200111000-00015 11699809

[ref33] Gresham F. M. Elliott S. N. (1990). Social skills rating system manual. Minneapolis, MN: NCS Pearson, Inc.

[ref34] Grusec J. E. Goodnow J. J. (1994). Impact of parental discipline methods on the child’s internalization of values: a reconceptualization of current points of view. Dev. Psychol. 30, 4–19. doi: 10.1037/0012-1649.30.1.4

[ref35] Jiang S. Dong L. Jiang C. (2020). Examining the link between economic strain and adolescent social behavior: roles of social bonds and empathy. J. Adolesc. 84, 1–10. doi: 10.1016/j.adolescence.2020.07.015, PMID: 32810758

[ref9002] Kerr D. C. R. Leve L. D. Harold G. T. Natsuaki M. N. Neiderhiser J. M. Shaw D. S.. (2013). Influences of Biological and Adoptive Mothers’ Depression and Antisocial Behavior on Adoptees’ Early Behavior Trajectories. J. Abnorm. Child Psychol. 41, 723–734. doi: 10.1007/s10802-013-9711-6 23408036 PMC3681846

[ref36] Kessler R. C. Üstün T. B. (2004). The world mental health (WMH) survey initiative version of the World Health Organization (WHO) composite international diagnostic interview (CIDI). Int. J. Method. Psychiat. Res. 13, 93–121. doi: 10.1002/mpr.168, PMID: 15297906 PMC6878592

[ref37] Kim H. K. Capaldi D. M. Pears K. C. Kerr D. C. R. Owen L. D. (2009). Intergenerational transmission of internalising and externalising behaviours across three generations: gender-specific pathways. Criminal Behav. Ment. Health 19, 125–141. doi: 10.1002/cbm.708, PMID: 19274624 PMC2715275

[ref38] Knafo A. Plomin R. (2006). Parental discipline and affection and children’s prosocial behavior: genetic and environmental links. J. Pers. Soc. Psychol. 90, 147–164. doi: 10.1037/0022-3514.90.1.147 16448315

[ref39] Kully-Martens K. Denys K. Treit S. Tamana S. Rasmussen C. (2012). A review of social skills deficits in individuals with fetal alcohol Spectrum disorders and prenatal alcohol exposure: profiles, mechanisms, and interventions: social skills in FASD and PAE. Alcohol. Clin. Exp. Res. 36, 568–576. doi: 10.1111/j.1530-0277.2011.01661.x, PMID: 22017360

[ref40] Layous K. Nelson S. K. Oberle E. Schonert-Reichl K. A. Lyubomirsky S. (2012). Kindness counts: prompting prosocial behavior in preadolescents boosts peer acceptance and well-being. PLoS One 7:e51380. doi: 10.1371/journal.pone.0051380, PMID: 23300546 PMC3530573

[ref41] Lee J. Y. Volling B. L. Lee S. J. (2021). Material hardship in families with low income: positive effects of Coparenting on fathers’ and mothers’ parenting and Children’s prosocial behaviors. Front. Psychol. 12:729654. doi: 10.3389/fpsyg.2021.729654, PMID: 34955959 PMC8696346

[ref42] Lenzi M. Vieno A. Perkins D. D. Pastore M. Santinello M. Mazzardis S. (2012). Perceived neighborhood social resources as determinants of prosocial behavior in early adolescence. Am. J. Community Psychol. 50, 37–49. doi: 10.1007/s10464-011-9470-x, PMID: 21932107

[ref43] Leve L. D. Anderson D. Harold G. T. Neiderhiser J. M. Natsuaki M. N. Shaw D. S.. (2022). Developmental profiles of child behavior problems from 18 months to 8 years: the protective effects of structured parenting vary by genetic risk. Dev. Psychopathol. 34, 1716–1730. doi: 10.1017/S0954579422000839, PMID: 35929354 PMC9899296

[ref44] Leve L. D. Griffin A. M. Natsuaki M. N. Harold G. T. Neiderhiser J. M. Ganiban J. M.. (2019). Longitudinal examination of pathways to peer problems in middle childhood: a siblings-reared-apart design. Dev. Psychopathol. 31, 1633–1647. doi: 10.1017/S0954579419000890 31439063 PMC6854293

[ref9003] Leve L. D. Neiderhiser J. M. Shaw D. S. Ganiban J. Natsuaki M. N. Reiss D. (2013). The Early Growth and Development Study: A Prospective adoption study from birth through middle childhood. Twin. Res. Hum. Genet. 16, 412–423. doi: 10.1017/thg.2012.126 23218244 PMC3572752

[ref9004] Li J. McMurray A. Stanley F. (2008). Modernity’s paradox and the structural determinants of child health and well-being. Health Sociol. Rev. 17, 64–77. doi: 10.5172/hesr.451.17.1.64

[ref45] Little R. J. A. (1988). A test of missing completely at random for multivariate data with missing values. J. Am. Stat. Assoc. 83, 1198–1202. doi: 10.1080/01621459.1988.10478722

[ref46] Liu C. Ji L. Chow S. Kang B. Leve L. D. Shaw D. S.. (2020). Child effects on parental negativity: the role of heritable and prenatal factors. Child Dev. 91, e1064–e1081. doi: 10.1111/cdev.13404, PMID: 32738072 PMC7722043

[ref47] Lüdecke D. (2018). Ggeffects: tidy data frames of marginal effects from regression models. JOSS 3:772. doi: 10.21105/joss.00772

[ref48] Luengo Kanacri B. P. Pastorelli C. Thartori E. Lunetti C. Di Giunta L. Bacchini D.. (2021). Longitudinal relations among maternal self-efficacy, maternal warmth, and early adolescents’ prosocial behavior. Parenting 21, 24–46. doi: 10.1080/15295192.2020.1777791, PMID: 33732095 PMC7959051

[ref49] Malhotra D. (2010). (when) are religious people nicer? Religious salience and the “Sunday effect” on pro-social behavior. Judgm. Decis. Mak. 5, 138–143. doi: 10.1017/S1930297500001005

[ref50] Malonda E. Llorca A. Mesurado B. Samper P. Mestre M. V. (2019). Parents or peers? Predictors of prosocial behavior and aggression: a longitudinal study. Front. Psychol. 10:2379. doi: 10.3389/fpsyg.2019.02379, PMID: 31695656 PMC6817951

[ref51] Marceau K. De Araujo-Greecher M. Miller E. S. Massey S. H. Mayes L. C. Ganiban J. M.. (2016). The perinatal risk index: early risks experienced by domestic adoptees in the United States. PLoS One 11:e0150486. doi: 10.1371/journal.pone.0150486, PMID: 27010541 PMC4807102

[ref52] Marceau K. Rolan E. Leve L. D. Ganiban J. M. Reiss D. Shaw D. S.. (2019). Parenting and prenatal risk as moderators of genetic influences on conduct problems during middle childhood. Dev. Psychol. 55, 1164–1181. doi: 10.1037/dev0000701, PMID: 30843708 PMC6533149

[ref53] Masten A. S. (2021). “Resilience in developmental systems: principles, pathways, and protective processes in research and practice” in Multisystemic resilience. ed. Ungar M. (New York: Oxford University Press), 113-C6.P173.

[ref54] Masten A. S. Lucke C. M. Nelson K. M. Stallworthy I. C. (2021). Resilience in development and psychopathology: multisystem perspectives. Annu. Rev. Clin. Psychol. 17, 521–549. doi: 10.1146/annurev-clinpsy-081219-120307 33534615

[ref55] Mazza J. R. S. E. Lambert J. Zunzunegui M. V. Tremblay R. E. Boivin M. Côté S. M. (2017). Early adolescence behavior problems and timing of poverty during childhood: a comparison of lifecourse models. Soc. Sci. Med. 177, 35–42. doi: 10.1016/j.socscimed.2017.01.039 28157567

[ref56] McNeil T. F. Cantor-Graae E. Sjöström K. (1994). Obstetric complications as antecedents of schizophrenia: empirical effects of using different obstetric complication scales. J. Psychiatr. Res. 28, 519–530. doi: 10.1016/0022-3956(94)90042-6, PMID: 7699611

[ref57] Meinhofer A. Witman A. Maclean J. C. Bao Y. (2022). Prenatal substance use policies and newborn health. Health Econ. 31, 1452–1467. doi: 10.1002/hec.4518, PMID: 35445500 PMC9177792

[ref58] Melby J. N. Conger R. D. (2001). “The Iowa family interaction rating scales: instrument summary” in Family observational coding systems: Resources for systemic research. eds. Kergin P. K. Lindahl K. M. (Mahwah, NJ: Erlbaum), 33–58.

[ref59] Memmott-Elison M. K. Carlo G. Maiya S. Roos J. (2021). A cross-ethnoracial comparison of objective and subjective neighborhood predictors of early adolescents’ prosocial behavior. Soc. Dev. 30, 767–785. doi: 10.1111/sode.12506

[ref60] Mujahid M. S. Diez Roux A. V. Morenoff J. D. Raghunathan T. (2007). Assessing the measurement properties of neighborhood scales: from psychometrics to Ecometrics. Am. J. Epidemiol. 165, 858–867. doi: 10.1093/aje/kwm040, PMID: 17329713

[ref9005] Natsuaki M. N. Leve L. D. Neiderhiser J. M. Shaw D. S. Scaramella L. V. Ge X.. (2013). Intergenerational transmission of risk for social inhibition: The interplay between parental responsiveness and genetic influences. Dev. Psychopathol. 25, 261–274. doi: 10.1017/S0954579412001010 23398764 PMC3576856

[ref61] Natsuaki M. N. Neiderhiser J. M. Harold G. T. Shaw D. S. Reiss D. Leve L. D. (2019). Siblings reared apart: a sibling comparison study on rearing environment differences. Dev. Psychol. 55, 1182–1190. doi: 10.1037/dev0000710, PMID: 30816723 PMC6533126

[ref62] Neiderhiser J. M. Marceau K. De Araujo-Greecher M. Ganiban J. M. Mayes L. C. Shaw D. S.. (2016). Estimating the roles of genetic risk, perinatal risk, and marital hostility on early childhood adjustment: medical records and self-reports. Behav. Genet. 46, 334–352. doi: 10.1007/s10519-016-9788-0, PMID: 27075497 PMC4860070

[ref63] Niccols A. Milligan K. Sword W. Thabane L. Henderson J. Smith A. (2012). Integrated programs for mothers with substance abuse issues: a systematic review of studies reporting on parenting outcomes. Harm Reduct. J. 9:14. doi: 10.1186/1477-7517-9-14, PMID: 22429792 PMC3325166

[ref64] O’Brien D. T. Kauffman R. A. (2013). Broken windows and low adolescent Prosociality: not cause and consequence, but co-symptoms of low collective efficacy. Am. J. Community Psychol. 51, 359–369. doi: 10.1007/s10464-012-9555-1, PMID: 23180238

[ref65] Padilla-Walker L. M. Carlo G. Memmott-Elison M. K. (2018). Longitudinal change in adolescents’ prosocial behavior toward strangers, friends, and family. J. Res. Adolesc. 28, 698–710. doi: 10.1111/jora.12362, PMID: 29144027

[ref66] Padilla-Walker L. M. Christensen K. J. (2011). Empathy and self-regulation as mediators between parenting and adolescents’ prosocial behavior toward strangers, friends, and family: prosocial behavior. J. Res. Adolesc. 21, 545–551. doi: 10.1111/j.1532-7795.2010.00695.x 29144027

[ref67] Pastorelli C. Zuffianò A. Lansford J. E. Thartori E. Bornstein M. H. Chang L.. (2021). Positive youth development: parental warmth, values, and prosocial behavior in 11 cultural groups. JYD 16, 379–401. doi: 10.5195/jyd.2021.1026, PMID: 34888590 PMC8651236

[ref9006] Pechtel P. Pizzagalli D. A. (2011). Effects of early life stress on cognitive and affective function: an integrated review of human literature. Psychopharmacol. 214, 55–70. doi: 10.1007/s00213-010-2009-2 PMC3050094 20865251

[ref9007] Pelham W. E. Dishion T. J. Tein J.-Y. Shaw D. S. Wilson M. N. (2017). What doesn’t work for whom? Exploring heterogeneity in responsiveness to the Family Check-Up in early childhood Using a mixture model approach. Prev. Sci. 18, 911–922. doi: 10.1007/s11121-017-0805-1 28550456 PMC5693624

[ref9008] Polderman T. J. C. De Geus E. J. C. Hoekstra R. A. Bartels M. Van Leeuwen M. Verhulst F. C.. (2009). Attention problems, inhibitory control, and intelligence index overlapping genetic factors: A study in 9-, 12-, and 18-year-old twins. Neuropsychol. 23, 381–391. doi: 10.1037/a0014915 19413451

[ref68] Preacher K. Curran P. Bauer D. (2023). Simple intercepts, simple slopes, and regions of significance in HLM 3-way interactions. Kristopher J. Preacher, viewed 12 August 8. Available at: http://quantpsy.org/.

[ref69] Putnam S. P. Rothbart M. K. (2006). Development of short and very short forms of the Children’s behavior questionnaire. J. Pers. Assess. 87, 102–112. doi: 10.1207/s15327752jpa8701_09, PMID: 16856791

[ref70] R Core Team (2022). R: A language and environment for statistical computing. R Foundation for Statistical Computing, Vienna. Available at: https://www.R-project.org/

[ref71] Robins L. N. (1981). National Institute of Mental Health diagnostic interview schedule: its history, characteristics, and validity. Arch. Gen. Psychiatry 38:381. doi: 10.1001/archpsyc.1981.01780290015001 6260053

[ref72] Robitzsch A. Grund S. Henke T. Robitzsch M. (2017). Miceadds: some additional multiple imputation functions, especially for mice. R package version 3.16–18 Available at: https://CRAN.R-project.org/package=miceadds

[ref73] Rothbart M. K. Ahadi S. A. Evans D. E. (2000). Temperament and personality: origins and outcomes. J. Pers. Soc. Psychol. 78, 122–135. doi: 10.1037/0022-3514.78.1.122 10653510

[ref74] Rothbart M. K. Ahadi S. A. Hershey K. L. Fisher P. (2001). Investigations of temperament at three to seven years: the Children’s behavior questionnaire. Child Dev. 72, 1394–1408. doi: 10.1111/1467-8624.00355, PMID: 11699677

[ref75] Rubin D. B. (1987). Multiple imputation for nonresponse in surveys. 1st Edn Hoboken, NJ: John Wiley & Sons, Inc.

[ref76] Saroglou V. Pichon I. Trompette L. Verschueren M. Dernelle R. (2005). Prosocial behavior and religion: new evidence based on projective measures and peer ratings. J. Sci. Study Religion 44, 323–348. doi: 10.1111/j.1468-5906.2005.00289.x

[ref77] Scarr S. McCartney K. (1983). How people make their own environments: a theory of genotype --> environment effects. Child Dev. 54:424. doi: 10.2307/1129703 6683622

[ref78] Shaw D. S. Sitnick S. L. Brennan L. M. Choe D. E. Dishion T. J. Wilson M. N.. (2016). The long-term effectiveness of the family check-up on school-age conduct problems: moderation by neighborhood deprivation. Dev. Psychopathol. 28, 1471–1486. doi: 10.1017/S0954579415001212 26646197 PMC4900930

[ref79] Shonkoff J. P. Boyce W. T. Levitt P. Martinez F. D. McEwen B. (2021). Leveraging the biology of adversity and resilience to transform pediatric practice. Pediatrics 147:e20193845. doi: 10.1542/peds.2019-3845, PMID: 33495367

[ref9009] Spinrad T. L. Eisenberg N. Valiente C. (2019). “Parenting and Children’s Prosocial Development”, in Handbook of Parenting, ed. Bornstein M. H. (London, UK: Routledge/Taylor & Francis Group), 91–121.

[ref80] Suchman N. E. Decoste C. Mcmahon T. J. Rounsaville B. Mayes L. (2011). The mothers and toddlers program, an attachment-based parenting intervention for substance-using women: results at 6-week follow-up in a randomized clinical pilot. Infant Ment. Health J. 32, 427–449. doi: 10.1002/imhj.20303, PMID: 22685361 PMC3370691

[ref81] Taylor L. K. Carlo G. (2021). Introduction to the special section: prosocial development in risky and vulnerable contexts. Int. J. Behav. Dev. 45, 289–292. doi: 10.1177/0165025421990759

[ref82] van Ham M. Manley D. (2012). Neighbourhood effects research at a crossroads. Ten challenges for future research introduction. Environ. Plan. A 44, 2787–2793. doi: 10.1068/a45439

[ref83] Wentzel K. R. McNamara C. C. (1999). Interpersonal relationships, emotional distress, and prosocial behavior in middle school. J. Early Adolesc. 19, 114–125. doi: 10.1177/0272431699019001006

[ref84] White I. R. Royston P. Wood A. M. (2011). Multiple imputation using chained equations: issues and guidance for practice. Statist. Med. 30, 377–399. doi: 10.1002/sim.4067, PMID: 21225900

[ref85] Williams J. H. G. Ross L. (2007). Consequences of prenatal toxin exposure for mental health in children and adolescents: a systematic review. Eur. Child Adolesc. Psychiatry 16, 243–253. doi: 10.1007/s00787-006-0596-6, PMID: 17200791

[ref86] Xiao S. X. Korous K. M. Hewitt S. L. Nguyen D. (2023). Family economic pressure and early adolescents’ prosocial behavior: the importance of considering the types of prosocial behavior. J. Adolesc. 95, 1220–1231. doi: 10.1002/jad.12196, PMID: 37211871

[ref87] Zhou Q. Eisenberg N. Losoya S. H. Fabes R. A. Reiser M. Guthrie I. K.. (2002). The relations of parental warmth and positive expressiveness to Children’s empathy-related responding and social functioning: a longitudinal study. Child Dev. 73, 893–915. doi: 10.1111/1467-8624.00446, PMID: 12038559

