# Supplementary material for: Examination of promotive and protective effects on early adolescent prosocial behavior through a bioecological lens
Source: Front Psychol. 2023 Nov 17;14:1280346. doi: 10.3389/fpsyg.2023.1280346 (PMC10690822; doi:10.3389/fpsyg.2023.1280346)
Supplement: Supplementary file 1 [file Data_Sheet_1.pdf]

Supplemental Table 1

Median, Skewness, and Kurtosis of Continuous Independent and Dependent Variables

Variables	Range	*M* or %	*SD*
Externalizing behaviors	-3.7-4.8	0.03	1.80
Internalizing behaviors	-3.5-5.4	0.02	1.56
Prosocial age 7	10.0-72.5	49.82	8.63
Prosocial age 11	1.0-10.0	8.31	1.59
Positive affectivity	3.4-7.0	5.86	0.53
Parental warmth	27.5-42.0	37.86	3.27
Neighborhood involvement	1.0-3.0	1.94	0.50
Importance of religion	1.0-4.0	1.98	0.94
Financial security	1.0-8.0	6.27	1.39
Neighborhood safety	2.0-4.80	4.56	0.31

Notes. Results are pooled estimates across 25 multiply imputed datasets.

Supplemental Table 2

Two-Way Interaction Models Predicting Prosocial Behavior at Age 11

	Risk factor = BF internalizing behaviors			Risk factor = BF externalizing behaviors			Risk factor = Prenatal substance use		
Variables	*b*	*SE*	95% CI	*b*	*SE*	95% CI	*b*	*SE*	95% CI
Risk factor	-0.54	0.72	-1.96, 0.88	-0.11	0.62	[-1.34, 1.22]	-2.10	2.11	[-6.26, 2.06]
Parental warmth	0.07	0.02	0.01, 0.13	0.07*	0.03	[0.01, 0.13]	0.05	0.04	[-0.02, 0.12]
Risk factor x Parental warmth	0.01	0.02	-0.03, -0.05	0.00	0.02	[-0.03, 0.03]	0.05	0.06	[-0.06, 0.16]

Notes. Results are pooled estimates across 25 multiply imputed datasets. BF = birth family; *b* = unstandardized beta coefficient; SE = cluster robust standard error; 95% CI = 95% confidence interval around unstandardized beta coefficient. **p* <.05, ***p* <.01

Supplemental Table 3

Three-Way Interaction Models Predicting Prosocial Behavior at Age 11

	Risk factor = BF internalizing behaviors			Risk factor = BF externalizing behaviors		
Variables	*b*	*SE*	95% CI	*b*	*SE*	95% CI
Risk factor	0.83	1.89	[-2.92, 4.58]	0.01	1.89	[-3.73, 3.76]
Household type	0.64	2.74	[-4.73, 6.01]	0.58	2.84	[-5.00, 6.17]
Parental warmth	0.07	0.06	[-0.06, 0.19]	0.06	0.07	[-0.06, 0.19]
Risk factor x Parental warmth	-0.03	0.05	[-0.13, 0.08]	0.00	0.05	[-0.10, 0.10]
Risk factor x Household type	-1.67	1.99	[-5.62, 2.28]	-0.16	2.04	[-4.20, 3.88]
Household type x Parental warmth	0.00	0.07	[-0.14, 0.14]	0.01	0.07	[-0.14, 0.15]
Risk factor x Parental warmth x Household type	0.04	0.05	[-0.06, 0.15]	0.00	0.05	[-0.10, 0.11]

Notes. Results are pooled estimates across 25 multiply imputed datasets. BF = birth family; *b* = unstandardized beta coefficient; SE = cluster robust standard error; 95% CI = 95% confidence interval around unstandardized beta coefficient.
$^{*}p < .05$, $^{**}p < .01$
